# Automated solid-phase peptide synthesis to obtain therapeutic peptides

**DOI:** 10.3762/bjoc.10.118

**Published:** 2014-05-22

**Authors:** Veronika Mäde, Sylvia Els-Heindl, Annette G Beck-Sickinger

**Affiliations:** 1 Institute of Biochemistry, Faculty of Biosciences, Pharmacy and Psychology, Universität Leipzig, Brüderstraße 34, D-04103 Leipzig, Germany

**Keywords:** automated synthesis, automation, lipidation, PEGylation, peptide drugs, solid-phase peptide synthesis, therapeutic peptides

## Abstract

The great versatility and the inherent high affinities of peptides for their respective targets have led to tremendous progress for therapeutic applications in the last years. In order to increase the drugability of these frequently unstable and rapidly cleared molecules, chemical modifications are of great interest. Automated solid-phase peptide synthesis (SPPS) offers a suitable technology to produce chemically engineered peptides. This review concentrates on the application of SPPS by Fmoc/*t*-Bu protecting-group strategy, which is most commonly used. Critical issues and suggestions for the synthesis are covered. The development of automated methods from conventional to essentially improved microwave-assisted instruments is discussed. In order to improve pharmacokinetic properties of peptides, lipidation and PEGylation are described as covalent conjugation methods, which can be applied by a combination of automated and manual synthesis approaches. The synthesis and application of SPPS is described for neuropeptide Y receptor analogs as an example for bioactive hormones. The applied strategies represent innovative and potent methods for the development of novel peptide drug candidates that can be manufactured with optimized automated synthesis technologies.

## Introduction

**Scheme 1:**
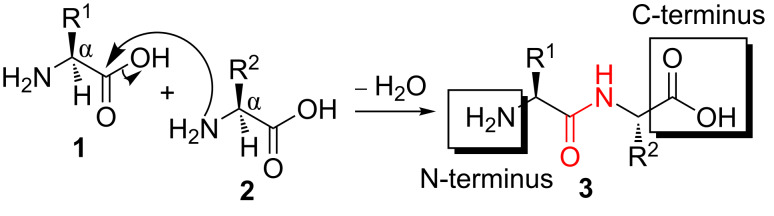
Formation of a dipeptide **3**. Reaction of the amino group of amino acid **2** with the carboxylic acid moiety of amino acid **1** leads to a mesomeric peptide bond (highlighted in red).

Peptides and proteins are involved in a large variety of biochemical processes and physiological functions. Peptides can consist of up to 50 amino acids and have generally no tertiary, three-dimensional structure compared to proteins [1]. In nature, the oligomers or polymers are assembled at ribosomes by aminoacyl-tRNAs (transfer ribonucleic acid) [2]. Basically, a condensation reaction of a carboxylic acid moiety with a functional amine of trifunctional α-amino acids leads to regioisomeric amide bond (peptide bond) formation (Scheme 1). The individual building blocks occur as L-enantiomers throughout living organisms in case of ribosomal synthesis and only 20 monomers are generally found in peptides and proteins with few rare exceptions. Those canonical amino acids vary in their side-chain functionality and possess different polarities that are important for their biological function.

Peptides can be biologically active hormones, neurotransmitters and neuropeptides, growth factors, signaling molecules and antibiotics. These diverse functions make peptides an interesting target on the pharmaceutical market. In terms of molecular weight, peptides bridge the gap between small molecule drugs (<500 Da) and protein-based pharmaceuticals (>5,000 Da) and enable a possible medication of incurable pathologies [3]. Diseases such as cancer, diabetes, obesity but also osteoporosis, cardiovascular diseases and inflammation can be treated by peptide-based drugs [4–5].

Within the last decades, the fast development of *omics* technologies such as genomics, proteomics and transcriptomics led to the identification of a great number of target peptides or proteins [6]. This trend successively offers new targets for peptide drugs that classical small organic molecules cannot cover [3]. Although small synthetic drugs are in general orally applicable owing to their high metabolic stability, capable to cross cell membranes and small in size, which simplifies their production and costs, they reveal considerable shortcomings. They show, for example, often moderate target potency and selectivity, which manifest in side-effects. In contrast, the strong and specific binding of peptides and proteins to their molecular targets can reduce the drug dose. This high selectivity leads to fewer side effects, which is considered as the greatest benefit of peptides and proteins over small molecules [7–8]. Moreover, small organic compounds are not able to address protein–protein interactions as their counterparts, the peptides/proteins [9].

Peptides share all superiorities of proteins but are significantly smaller in size and hence, easier and cheaper to synthesize using chemical strategies [5]. Thereby, they provide a vast perspective for novel drug design. Table 1 summarizes valuable virtues and pivotal shortcomings of therapeutic peptides compared to traditional small organic molecules. The high potency and selectivity of peptides are of great advantage for drug development [4]. The metabolization leads to non-toxic degradation products, which, combined with their high specificity, goes along with low adverse effects. Furthermore, peptides do not tend to interact with other drugs and exhibit a more predictable in vivo behavior owed to their biochemical nature [7]. The extended size and the tremendous biological and chemical diversity of peptides opposed to small organic drugs opens targets for multiple applications [10]. In the last decades, the production of therapeutic peptides has been revolutionized by new methods and strategies for automated approaches, which simplifies peptide manufacturing. Combined with the mentioned advantages of peptide-based drugs, their application as novel biopharmaceuticals is pushed forward.

**Table 1:** “Drugability” attributes of peptide therapeutics compared with small molecules.

Virtues	Drawbacks

high activity, specificity and selectivity	low metabolic stability
few side-effects	short circulating half-life
no/less toxic degradation products	rapid body clearance if <5,000 Da [9]
no drug–drug interactions	low (oral) bioavailability, mainly parenteral administration needed
more in vivo predictability	poor membrane permeability
large interaction sides	sometimes reduced water solubility
biological and chemical variety	risk of immunogenic effects
able to target protein–protein interactions	expensive synthesis

Within the last years, the global market for peptide therapeutics expanded nearly twice as fast as overall drugs [7]. Up to now, nearly 70 peptide drugs were approved by the US Food and Drug Administration (FDA) and reached the medicinal market [11]. In addition, many peptides are currently in clinical (>150) and advanced preclinical (>400) phases, exemplifying the urgent demand of peptides for various indications [8]. In 2005, the market for peptide drugs covered 8 billion EUR and was estimated to reach 11.5 billion EUR in 2013 [5]. The market growth rate has been projected to be over 10% per year. To date, 4% of overall approved pharmaceuticals are peptide hormones or derivatives [12].

Besides this success story, there are limitations restricting the use of peptides as drugs (Table 1). Notably, their low bioavailability owing to proteolytic degradation by enzymes of the intestine, blood and cell plasma leads to short circulating half-lives [13]. Depending on their size, peptides are excreted by kidneys (renal clearance) or liver (hepatic clearance) within minutes [5,9]. Nevertheless, their ability to pass through membranes and the urgent need of alternative, more comfortable administration routes as the commonly used parenteral (subcutaneous, intramuscular and intravenous) application, have prompted further research in this field [14]. Therefore, methods to prolong peptide stability are of great interest.

Here, we highlight the importance of automated solid-phase peptide synthesis (SPPS) in the process of peptide modification. Principles of chemical synthesis of peptides are covered with a focus on Fmoc (9-fluorenylmethoxycarbonyl)/*t*-Bu (*tert*-butyl)-based solid-phase peptide synthesis. Recent advances in automation devices are described, with attention to the comparison between conservative SPPS robots and microwave-assisted automated SPPS. Moreover, strategies for modulating peptide stability with an emphasis on lipidation and PEGylation are characterized. Last, the syntheses of selected peptide hormones are presented exemplarily.

## Review

### Chemical synthesis of peptides and its automation

#### Solid-phase peptide synthesis – the way from homogeneous to heterogeneous synthesis

In the past, pioneering of Emil Fischer at the beginning of the 20^th^ century [15] and du Vigneaud in 1953 [16] have made the synthesis of peptides possible, as at that time, they were relatively unknown biomolecules. Fischer synthesized the first dipeptide, called glycylglycin, and coined the term “peptide” [15]. Fifty years later, du Vigneaud developed a strategy for the production of a polypeptide. For the synthesis of the polypeptide hormone oxytocin, organic protecting groups were introduced to trifunctional amino acids [17] in order to ensure specific amide-bond formation [16]. The principle of peptide synthesis in homogenous solution is based on the reversible blocking of the carboxylic acid function of the C-terminal amino acid and the amino group of the N-terminal amino acid. In addition, activation of the free carboxy group of the N-terminal amino acid is necessary to obtain the peptide bond. For this approach, all peptide intermediates have to be isolated and purified before they can be used for further reaction steps. Although this assures a good quality control, it is a very time-consuming and a technical-demanding process [18]. This manifests especially at larger and more complex peptides, for which the protected fragments often tend to be rigid and insoluble [19].

**Scheme 2:**
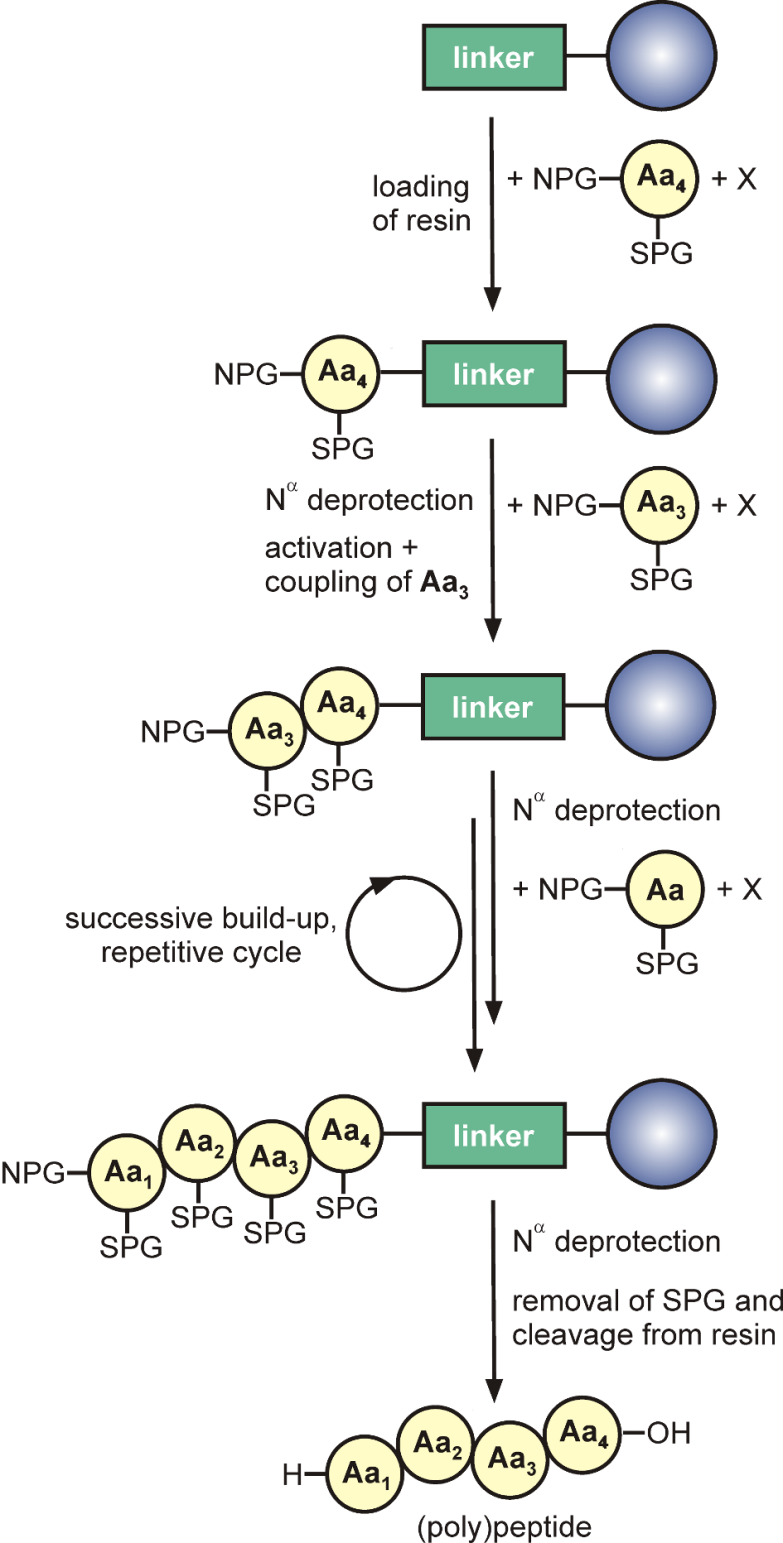
Peptide assembly by SPPS, exemplarily shown for a tetrapeptide. First, the C-terminal amino acid is coupled to the linker. The peptide chain will be elongated by repeating a cycle of 1) deprotection of NPG, 2) activation of the carboxy group and 3) coupling. At the end of the synthesis, the protecting groups will be cleaved and the desired peptide obtained. NPG: N^α^-protecting group, X: activator, SPG: side-chain protecting group, Aa: amino acid.

These disadvantages in the synthesis of peptides led to the revolutionary inception of a completely different strategy. In 1963, Bruce Merrifield published the synthesis of a tetrapeptide, which was assembled under heterogeneous conditions from the C- to the N-terminus on a polymeric solid “resin” [20]. The method was named solid-phase peptide synthesis and accounts for a peptide construction between two phases, an insoluble solid support and liquid soluble reagents [21]. Here, the first amino acid is coupled for the time of the synthesis with its carboxylic acid terminus to a resin that consists of polymer particles and protects the C-terminus from side reactions. In order to overcome aggregate formation, a distinct short organic linker is interposed between the amino acid and the solid support, which also determines the C-terminal modification of the synthetic peptide [22]. In addition, the N^α^-amino group and reactive side-chain moieties of trifunctional amino acids have to be blocked. N^α^-modifications serve as temporary protecting groups and can be removed specifically after each successful coupling step, whereas the side-chain protecting groups and the resin ensure a permanent protection against unwanted side reactions [20]. Moreover, the relatively inert carboxy group has to be activated by a special auxiliary to increase the electrophilicity [23]. After loading of the resin, the N-terminal protecting group of the first amino acid can be removed and the next activated building block can be coupled. These alternating steps of N^α^-deprotection, activation and coupling are repeated until the desired peptide chain is obtained. Following, the N^α^-protecting group of the N-terminal amino acid has to be deprotected and conditions to remove both, side-chain protecting groups and the peptide from the resin, have to be used (Scheme 2) [20]. The last step of SPPS should be performed in the presence of scavengers to trap highly reactive carbocations that are formed during the cleavage procedure and that might react with the peptide to form unwanted byproducts [24]. The crude product can be easily separated from the resin and purified by standard analytical methods such as the diverse chromatographic techniques. Their strong development with excellent improvement in separation of similar components was a major prerequisite for the success of SPPS, both with respect to analytics and preparative purification [25]. Furthermore, high-quality mass spectrometry (MS) with soft ionization techniques such as MALDI–TOF (matrix-assisted laser desorption ionization – time of flight) and ESI (electrospray ionization) MS allows nowadays rapid and clear identification of the respective product and all byproducts [9].

This heterogeneous synthesis technique offers great advantages. Certainly, the most important benefit of SPPS is the feasibility of carrying out all reactions in a single vessel. Following a coupling step, unreacted reagents and byproducts can be easily removed by washing, which makes purification of intermediates redundant. Based on the use of excess amounts of reactants, high coupling yields can be obtained and the incorporation of difficult sequences and modifications to the polymer are enabled. Moreover, the reaction cycles are very short compared to solution synthesis, which allows faster manufacturing [20]. Additionally, the solid-phase concept is not only an elegant way to build up peptides but also other oligomers such as polyamides [26], polynucleotides [27] and polysaccharides [28]. This method simplified the chemical synthesis of peptides and allowed the automation of the process [24], which has led to a breakthrough of SPPS and the establishment as one major technique for therapeutic peptide production [8,19].

#### Important selections in Fmoc/*t*-Bu orthogonal protecting-group strategy

**Figure 1:**
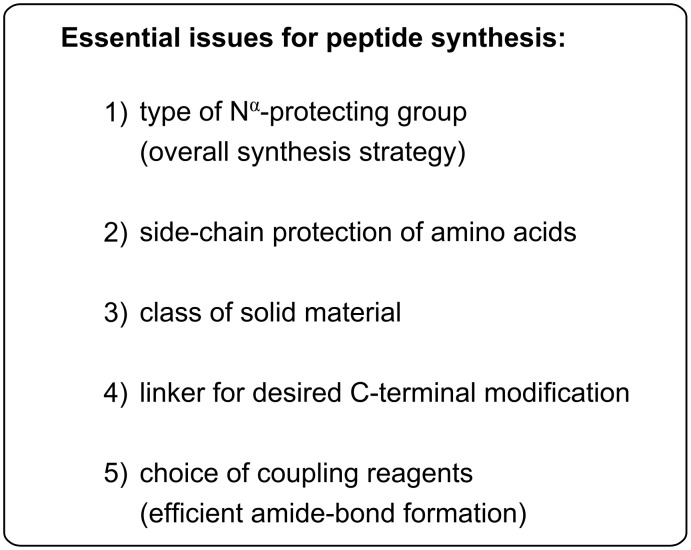
Five issues that have to be resolved prior to peptide synthesis.

**Scheme 3:**
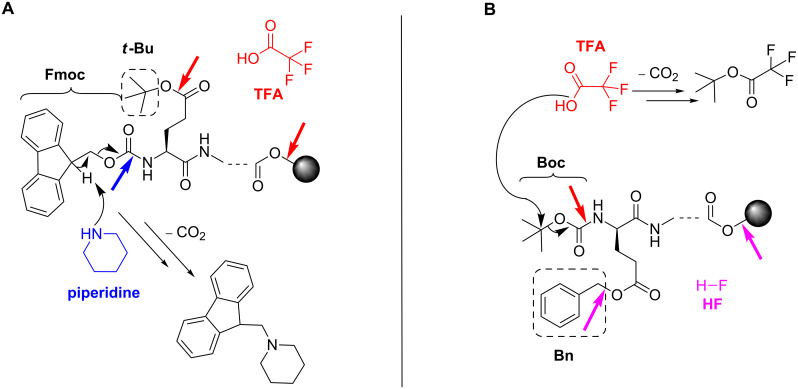
Fmoc/*t*-Bu (A) and Boc/Bn (B) protecting-group strategies applied in SPPS. (A) The Fmoc-group is removed by β-elimation through piperidine and *t*-Bu is released by acidolysis with TFA. (B) Cleavage of protecting groups with TFA and HF occurs by acidolysis. Removal reactions of the respective protecting groups are illustrated. Blue arrows indicate basic and red/purple arrows indicate acidic conditions. Dashed boxes stress the COOH-side-chain protecting group of glutamic acid exemplarily, used in each strategy.

**Protection of amino- and side-chain functionalities:** Protecting organic functionalities against side reactions and thus, formation of undesired chemical bonds is mandatory for SPPS (Figure 1). Requirements for appropriate protecting groups are the simple incorporation into the desired molecule, a high stability against various conditions as well as easy and safe removal [29]. For SPPS, two major protecting groups for the N^α^-amino function have been established: Boc (*tert*-butyloxycarbonyl) [30] and Fmoc [31]. The initial method applied by Merrifield was based on the use of the Boc group as temporary protecting group for the amino function and Bn (benzyl) or related protecting groups for the side chains of trifunctional amino acids. Usually, Boc can be removed by treatment with TFA (trifluoroacetic acid), whereas Bn deprotection requires strong acids such as HF [32]. Hence, this Boc/Bn protecting-group strategy is based on graded acid lability of permanent (also including the linkage to the solid support) and transient protecting groups (Scheme 3B). Whilst the Boc group has been used exclusively during the first years of SPPS, the introduction of the Fmoc-group [31] opened the path for a novel, more variable synthesis concept. Here, the Fmoc-group, which can be removed by basic conditions, serves as temporary N^α^-protecting group [33]. Side-chain protecting groups as *t-*Bu and the linkage of the peptide to the resin are unstable towards TFA-treatment [34–35] (Scheme 3A). Nowadays, both protecting group strategies are used for the synthesis of peptides and both methods can be applied for automated synthesis.

Nevertheless, the Fmoc/*t*-Bu protecting-group approach offers the great advantage of orthogonality. This concept [36] enables the selective removal of the protecting groups using completely different chemical conditions and cleavage mechanisms, which ensures milder overall reactions [37]. Although the Boc/Bn protecting strategy is accepted to be more suitable for the synthesis of difficult sequences and an aggregation of the peptide by repetitious TFA treatment can be prevented [38], the advantages of the Fmoc/*t*-Bu strategy are notable. The orthogonality is the main benefit of the Fmoc-based concept allowing a higher flexibility for complex strategies during synthesis. Moreover, the Fmoc strategy does not require the use of special vessels that have to be stable towards the corrosive and toxic HF and in some cases, the repetitive TFA acidolysis for Boc deprotection could have an impact on sensitive peptide bonds and acid-catalyzed side reactions [39]. And, since it is no orthogonal strategy, the Bn removal always leads to Boc deprotection.

**Figure 2:**
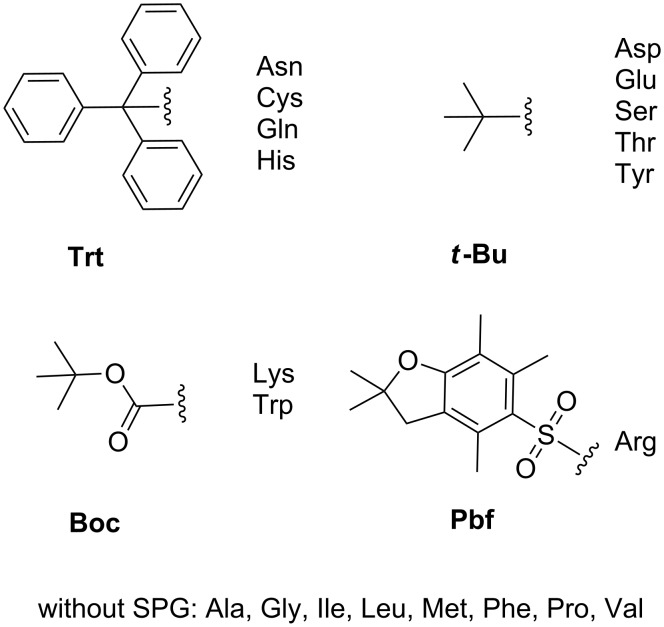
Commonly applied amino acid side chain protecting groups (SPG) in Fmoc/*t*-Bu-strategy. Trt: trityl, Pbf: pentamethyl-2,3-dihydrobenzofuran-5-sulfonyl.

A tremendous diversity of side-chain protection groups for trifunctional amino acids has been evolved since the development of SPPS more than 50 years ago. Proteinogenic amino acids contain different functional groups: amino, carboxyl, hydroxy, thio, pyrrolidinyl, imidazolyl, guanidinyl, amido and indolyl. Basically, every amino acid containing chemically reactive side chains has to be equipped with a protecting group during peptide assembly by SPPS in order to prevent side reactions and the formation of byproducts. In Figure 2, commonly used protecting groups in Fmoc/*t*-Bu-SPPS are illustrated for standard amino acid monomers. These protecting groups are orthogonal to the base-labile Fmoc-group and can be cleaved by highly concentrated TFA solutions. In addition to these examples there is a number of diverse orthogonal protecting groups commercially available. They will have to be used, if peptides are modified additionally and they are cleaved under specific conditions as, e.g., hydrazine (Dde (1-(4,4-dimethyl-2,6-dioxocyclohex-1-ylidene)-3-ethyl) group [40]), very low concentrated acids (Mmt (monomethoxytrityl) group [41]), palladium-catalyzed cleaving conditions (Alloc (allyloxycarbonyl) group [42]) or UV light (Nvoc (6-nitroveratryloxycarbonyl) group [43]). For a precise overview, the review of Isidro-Llobet 2009 and detailed manuals of major companies are recommended [44–46].

**Optimal resins and linkers for peptide synthesis:** The solid phase has to meet a number of requirements to be suitable for peptide synthesis. It has to be insoluble in all solvents, chemically and physically resistant and mechanically stable to allow filtration. Since peptide synthesis takes place mainly in the interior of the solid matrix, appropriate solvation, low cross linking for good accessibility and good swelling properties are very crucial. The small resin beads can enlarge up to six times of their original volume in organic solvents. Moreover, the polymer needs to have a functional group for coupling the linker [20–21,24]. The first solid polymer for peptide synthesis was presented by the SPPS founder Merrifield in 1963, a copolymer consisting of styrene and cross-linked divinylbenzene [20]. At present, there are mainly three classes of solid carriers: traditional polystyrene (PS), polyethylene glycol (PEG)-functionalized PS (such as TentaGel-supports [47]) and pure PEG-based resins such as PEGA resin [48] and ChemMatrix [49]. Shelton et al. recently published a collection of commonly used resins, together with their individual swelling and loading (is defined by the equivalents of amino acid in mmol/g, which can be attached to the resin) properties [50]. With respect to PEG-functionalized linkers, peptide synthesis yields can be improved by appropriate PEG units, loading and cross linking leading to elevated solubility and decreased intra- and intermolecular aggregation of the growing polypeptide [50].

The linker represents the reversible connection between the solid support and the assembling peptide. It determines the loading of the resin, the distance between resin and peptide, chemical conditions for coupling and release and most importantly, the C-terminal functionality of the synthetic peptide. In most cases, the peptide is released as acid or amide because these are naturally occurring C-terminal functionalities. Additionally, the C-terminus can be modified as hydrazide, alcohol, aldehyde, thioester and many more [22,50]. Furthermore, there are linkers that enable the synthesis of partially and fully protected peptides such as the 2-chlorotrityl resin [51] or the Sieber amide resin [52]. Consequently, the choice of resin and linker is based on the complexity of the desired peptide sequence, and the chemical reaction conditions as well as the peptide C-terminal modification.

**Figure 3:**
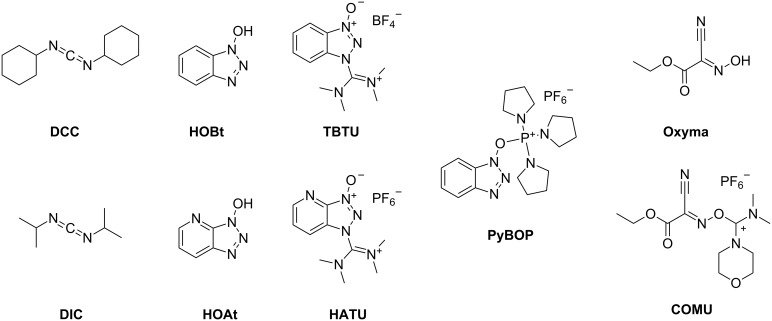
Selected coupling reagents for SPPS.

**Activation and coupling reagents:** In order to form peptide bonds by SPPS, the free carboxy terminus has to be transformed into an active, more electrophilic species. Therefore, carbodiimide-based coupling reagents such as DCC (*N*,*N*'-dicyclohexylcarbodiimide) [20] and DIC (*N*,*N*'-diisopropylcarbodiimide) [53–54] were used and considered as the major activators for many decades. The susceptibility of this efficient activator to racemize led to the required development of racemization suppressants such as the most popular HOBt (1-hydroxybenzotriazole) [55]. HOBt traps the *O*-acylisourea, an intermediate, which tends to racemization, and forms the activated species. Nowadays, a great variety of coupling reagents are commercially available reaching from traditional carbodiimides (DCC, DIC), classical auxiliary nucleophiles (HOBt, HOAt (1-hydroxy-7-azabenzotriazole) [56]) to uronium reagents (HATU (*N*-[(dimethylamino)-1*H*-1,2,3-triazolo-[4,5-*b*]pyridin-1-ylmethylene]-*N*-methylmethanaminium hexafluorophosphate *N*-oxide) [56], TBTU (*N*-[(1*H*-benzotriazol-1-yl)(dimethylamino)methylene]-*N*-methylmethanaminium tetrafluoroborate *N*-oxide) [57]), and phosphonium salts (PyBOP (benzotriazol-1-yloxytri(pyrrolidino)phosphonium hexafluorophosphate) [58]) (Figure 3). In 2009, Oxyma (ethyl 2-cyano-2-(hydroxyimino)acetate) [59–60] was introduced as a novel additive for DIC-mediated peptide-bond formation as an alternative to the well-established HOBt. This introduction was essential because of the inherent explosive potential of HOBt. Moreover, COMU ((1-cyano-2-ethoxy-2-oxoethylidenaminooxy)dimethylamino-morpholino-carbenium hexafluorophosphate) [61] has been identified as a safe and potent Oxyma-based uronium salt. Dependent on the bulkiness of the amino acids to be coupled and the chemical conditions such as its solubility and its stability, the decision of the proper coupling reagent, offering enhanced reactivity by simultaneous reduction of epimerization, is of high relevance.

#### Automated and multiple synthesis by SPPS

The main goal of the invention of SPPS was, from the onset, to open the path for a faster, simpler and a particularly more automated mode of operation for chemical peptide synthesis [21,24]. Peptide assembly on solid support can be performed simultaneously in different reaction vessels to construct diverse peptide sequences in a time-saving manner. During synthesis, Fmoc deprotection and washing are carried out for all peptides with the same solutions, whereas the coupling of amino acids is done individually but at the same time. In 1985, Houghten et al. reported the parallel synthesis of different peptides, which was performed at polystyrene-based polymers welded in a polypropylene network [62]. Each so called 'tea bag' thereby reflects one independent peptide. In this method, collective deprotection and washing are carried out in a polyethylene (PE) bottle, whilst for coupling the bags are separated according to their next amino acid to be attached and reacted in separate containers. Coding allows the identification and respective sorting of the tea bags. After synthesis completion, peptides are individually cleaved from the polymer support [63]. A further method of multiple parallel SPPS is the synthesis on amino-functionalized PE rods (pins) [64]. Here, a small amount of peptides is synthesized in a microtiter plate format. Coupling is performed in corresponding plates containing individual amino acid solutions and the collective washing occurs in tanks. Following the synthesis, the peptides remain on the polymer carrier enabling a fast and parallel screening for antibody binding by ELISA (enzyme-linked immunosorbent assay) (‘PepScan’). Although quality control is not possible using this strategy, it is suitable for fast epitope mapping. In addition, SPPS can be carried out on cellulose papers, in which reagents are spotted onto porous membranes [65]. The typical reactions proceed only in the volume that has been infused into the solid pores. Again, coupling and deprotection reactions as well as washing steps take place simultaneously. The immobilized peptides can finally be tested for specific enzyme reactions (peptide arrays [65]). All these methods are variations of the solid-phase synthesis concept [63].

But Bruce Merrifield had another vision. He wanted peptide synthesis to be performed in single reaction vessels that are placed in a special reactor block [20]. The instrument should be equipped with a plumbing system in order to realize pumping, mixing and removal of solutions. Moreover, the automated peptide robot should contain reservoirs for all chemicals (amino acids, solvents and activators) and ensure adequate delivery of the solutions. The first liquid-handling apparatus performing SPPS of this type was described in 1965 [24]. The improvement of chemical reactions, solid supports, linkers and especially the development of the Fmoc-based SPPS-strategy [31,37] have contributed to simplification and many advancement of following instruments. Today, peptide synthesizers can be purchased from more than 15 companies. Pedersen and Jensen recently reviewed commonly used devices for fully automated single and parallel Fmoc-SPPS [66]. Peptide synthesizers mainly differ in their type of solution transfer, mixing, synthesis scale and some special features such as automated monitoring (e.g., via chromophors [67] or conductivity [68]), microwave heating, capability for inert atmosphere and automated peptide cleavage [65]. The systems are mostly based on filtration by vacuum or pressure application [69], or centrifugation [70] and can work in a batch-wise or a continuous-flow modus. The decision for a peptide synthesizer has to be made according to the intended application and thus, the scale (necessary amounts), the type of chemistry (Fmoc or Boc-strategy) and the number of reaction tubes (plates or vessels). The decision should also be influenced by the length and complexity of the desired peptides.

Difficult and larger peptides often lead to impurities, synthesis termination and finally low yields owing to inter- and intramolecular aggregation and sterical hindrance. To reduce these effects, microwave-assisted instruments have been evolved [71]. In 1992, Yu and coworkers reported for the first time that microwave irradiation in combination with SPPS leads to enhanced reaction rates and hence, to a higher quality of the crude peptides [72]. Microwave energy is capable of activating any molecule containing a dipole moment, which is reflected in rapid heating on a molecular level [73]. The strength of the heating is influenced by solvents, reactants, sample volumes and the mode of mixing. It has to be noted that an optimization of temperature is mandatory in order to avoid racemization and side reactions [74].

#### Application of automated SPPS for drug development

The described method of chemical synthesis of peptides on the solid phase and particularly, its outstanding potential for automation, have led to routine methods in the development of novel pharmaceuticals. In principle, there are two approaches for drug design: rational and combinatorial methods. Whilst the rational procedure is a lead structure-oriented process, there is typically little knowledge about the evaluated biological system in combinatorial methods. In order to identify a lead compound of a relative unknown system, numerous molecules (peptides) have to be produced in parallel by a combination of building blocks creating a peptide library [75]. The first parallel synthesis of hundreds of peptides was published by Geysen et al. in 1984 [64]. Here, a series of peptide epitopes was synthesized on a multipin instrument and used for an enzymatic assay. Peptide libraries can be created by directed parallel synthesis or complex peptide mixtures and identified by iterative processes or position screenings [63]. A polymer-bound peptide library can be produced by the 'one bead one compound' concept [76]. Here, each solid particle serves as a compartment to assemble an individual peptide, which can be sequenced for identification of the lead structure if it has shown an effect (“hit”).

#### Industrial synthesis of peptides and alternative production methods

The successful automation of peptide synthesis led to a breakthrough on the peptide therapeutics market and vice versa. Thus, solid-phase synthesis is presently, besides the solution technique, one of the major procedures for peptide manufacturing [10]. The great efforts in improving linkers, protecting groups, resins etc. provided access for the synthesis of larger peptides and even proteins. But nevertheless, the sequential and convergent production for therapeutic applications is often the only possibility for manufacturing peptides, which are larger than 50 amino acids [77–78]. This method is based on the independent synthesis of fully protected linear peptide fragments being selectively condensed in solution to obtain the desired polypeptide. The condensation can occur via chemoselective ligation techniques such as native chemical ligation (NCL), expressed protein ligation (EPL), Staudinger ligation or click reaction [8,79].

Recently, peptide drugs as the pharmaceuticals Enfuvirtide, Eptifibitide and Bivalirudin have been manufactured in multikilogram scale [10,19]. Enfuvirtide (T-20/ Fuzeon^®^), for example, is an efficient membrane fusion inhibitor for HIV treatment consisting of 36 amino acids [80]. The large-scale bulk production of Fuzeon^®^ is performed by solution-phase fragment condensation from three side-chain protected intermediates synthesized at chlorotrityl resin [51]. Despite the many steps and high costs for this synthesis strategy, it is much more time-efficient due to the repetitive and semi-automated processes when compared to classical solution production [81]. Furthermore, the procedure yields in very high purities of the final peptide, which surely would not have been possible by any other technique.

In addition to solid and solution-phase synthesis, there are some other possibilities to produce these important molecules. Salmon calcitonin, human glucagon and human insulin are polypeptides being commercially produced by recombinant expression [10]. In general, the quality of chemically synthesized peptide therapeutics is comparable to recombinantly or enzymatically produced compounds.

The success story of SPPS, which has been going on for 50 years now, has shown that these molecules can be built up with a great variety of methods. The appropriate procedure strongly depends on the application (lead-structure discovery, biological investigations, potential drug candidate) of the desired peptide.

### Combination of automatic and manual SPPS to obtain therapeutic peptides

The majority of peptides are hormones being responsible for a broad scope of physiological functions. Here, we highlight two successful strategies to modify chemical properties in order to influence pharmacodynamic and -kinetic profiles – lipidation and PEGylation. As an example, modern concepts of SPPS-assisted, selective derivatization is described for neuropeptide Y (NPY) receptor ligands for therapeutical and analytical applications.

#### Modifications of therapeutic peptides to extend their half-lifes

As summarized in Table 1, natural peptides suffer from fast proteolytic degradation and body clearance. Furthermore, possible reduced water solubility restricts their drugability. In the last years, remarkable efforts have been made to modulate the bioavailability of peptides. Basically, delivery challenges of peptide drug candidates can be overcome by chemical modification or innovative formulation techniques such as the integration of peptides into particles, gels or liposomes [14,82–83]. Recently, the great methodical repertoire for extending the half-lifes of biological active peptides by covalent chemical approaches has been reviewed [8]. These methods include peptide sequence modifications by non-proteinogenic amino acids such as D- [84] or *N*-methylated [85–86] monomers or general truncation or mutation of biologically not relevant positions creating peptide analogs [87]. Likewise, backbone manipulation by partial or complete cyclization [88] as well as incorporation of peptide bond mimetics [89] can help to increase stability towards proteases. Peptide stability can also be optimized by blocking their respective termini through N-terminal acylation and C-terminal amidation [5]. Apart from this, metabolically unstable peptide drugs can be optimized by the covalent attachment of fatty acids (lipidation) or methoxy polyethylene glycol (PEG) polymers (PEGylation) [14]. These two strategies are based on substantially different mechanisms, which can lead to a remarkable increase of the potential utility of peptides as pharmaceuticals.

**Lipidation of peptides:** In general, the half-life extension of peptides by lipidation is obtained by an increased binding to albumin, which is the most abundant protein (6 mM in blood plasma [90]) within the extracellular fluid [91]. Human serum albumin (HSA) is a fundamental carrier of non-esterified free fatty acids as well as multiple other endogenous ligands and drugs in the blood. Early structural studies described a spherical folding of albumin [92] allowing electrostatic interaction between the carboxylate anion of fatty acids and positively charged residues of albumin [93]. Furthermore, hydrophobic interactions were shown to contribute to albumin binding in a cooperative effect [94], which means that albumin binding significantly increases until an appropriate fatty acid chain length is reached. In 1998, seven binding sites of this multifunctional transport protein were identified by Curry et al. using X-ray crystallographic studies [95]. Later on, they were distinguished in high and low affinity binding sites [96]. These properties were transferred for the first time to an important peptide hormone with high propensity to degrade in 1995 [91]. Here, insulin was acylated with saturated fatty acids containing 10 to 12 carbon atoms at the B-chain using the ε-group of the lysine^29^. In this study, the authors determined an increased albumin affinity of lipidated insulin variants depending on the number of carbon atoms by interaction studies with immobilized HSA. Moreover, they were able to show a sustained lowering effect of blood glucose, demonstrating a prolonged action profile of the acylated conjugates. Thus, the extended action was proposed to be facilitated by serum albumin binding, which leads to gradual peptide release and an prolonged circulation time [91]. Since then, many biological relevant peptides and proteins were chemically modified by fatty acid acylation [97].

The synthesis of these lipidated peptides can be performed by amidation, *S*- or *O*-esterification as well as thioether or -sulfide formation. Owing to the strength of the covalent bonds, amidation and *O*-esterfication are preferred over the other strategies [97]. Chemical synthesis of lipidated peptides is mostly performed by SPPS using the Fmoc/*t*-Bu strategy allowing for selective and efficient modification. Fatty acids can be incorporated into the peptide sequence at the N-terminus [98], at lysine [99] or cysteine side chains [100] and by esterification [101]. A detailed overview of chemical approaches to obtain lipidated peptides containing examples for each strategy is given by Zhang et al. [97]. In many cases, the on-resin lipidation is carried out at the lysine side chain [102–103]. Therefore, the peptide backbone can be synthesized by automated SPPS and the N^ε^-group of the lysine that should be modified, is protected specifically by a side-chain protecting group that is orthogonal to the Fmoc group. Acid-labile groups as Mmt [41] and Mtt (4-methyltrityl) [104] (classical cleavage with 1% TFA in DCM (dichloromethane)), base-labile groups as ivDde (1-(4,4-dimethyl-2,6-dioxocyclohex-1-ylidene)-3-methylbutyl) [105] and Dde [40] (deprotection with 2% hydrazine in DMF (*N*,*N*-dimethylformamide)) or the Alloc group [42] (cleavage with catalytically amounts of Pd(PPh)_3_ under inert conditions) are recommended. A selective removal of the side-chain protecting group enables specific amide-bond formation by the reaction of an activated carboxylic group of the fatty acid with the N^ε^-group of the lysine. The introduction of a glutamyl spacer can be helpful in order to increase the solubility of the drug candidates [99,106]. Lipidation of peptide hormones has led to great success with the myristoylated insulin analog insulin determir (Levemir^®^) [102] and the palmitoylated incretin mimetic GLP-1 (glucagon-like peptide 1) variant liraglutide (Victoza^®^) [106] (Table 2). Both peptide drugs reached market approval due to their prolonged blood glucose-lowering effects making them valuable for diabetes treatment. In 2011, a lipidated analog of PP (pancreatic polypeptide), a gut hormone that is known to mediate satiety, was developed. It showed an improved bioavailability demonstrated in a prolonged action in decreasing food intake in mice [99]. Apart from albumin interaction, there is also the possibility to increase peptide stability by direct fusion with HSA. One example of this effect is the GLP-1 analog CJC-1131. It contains a covalently attached albumin moiety and a D-amino acid at a labile position to obtain increased metabolic stability [107].

**Table 2:** PEGylated and lipidated peptide and protein drugs on the market, as stated by the FDA [11].

compound	product name (company)	indication	market entry (FDA approval)

PEGademase bovine	Adagen^®^ (sigma-tau)	severe combined immunodeficiency disease	1990
PEGaspargase	Oncaspar^®^ (sigma-tau)	acute lymphoblastic leukemia	1994
PEGinterferon α-2b	PEGIntron^®^ (Merck)	hepatitis C; also in combination with ribavirin	2001
PEGinterferon α-2b	Sylatron^®^ (Merck)	malignant melanoma	2001
PEGfilgrastim	Neulasta^®^ (Amgen)	neutropenia during chemotherapy	2002
PEGinterferon α-2a	Pegasys^®^ (Hoffmann-La Roche)	hepatitis C; also in combination with ribavirin	2002
PEGvisomant	Somavert^®^ (Pfizer)	acromegaly	2003
PEGaptanib	Macugen^®^ (Pfizer)	wet age-related macular degeneration	2004
mPEG-epoetin β	Mircera^®^ (Hoffmann-La Roche)	symptomatic anaemia associated with chronic kidney disease	2007
Certolizumab PEGol	Cimzia^®^ (UCB)	rheumatoid arthritis and Crohn's disease	2008
PEGloticase	Krystexxa^®^ (Savient Pharmaceuticals)	refractory chronic gout	2010

Insulin detemir	Levemir^®^ (Novo Nordisk)	diabetes mellitus	2005
Liraglutide	Victoza^®^ (Novo Nordisk)	diabetes mellitus type 2	2010

**PEGylation of peptides:** Another elegant way to modulate pharmacokinetic and -dynamic properties of peptide drugs is the formation of drug–polymer conjugates by PEGylation. PEGylation is the covalent modification of peptides with methoxy polyethyleneglycol polymer units of an averaged molecular weight. PEG itself is known to be amphiphilic, non-toxic, little immunogenic, non-antigenic and highly soluble [108]. These beneficial attributes are conveyed to the peptide by covalent attachment leading to an increased size, which lowers renal ultrafiltration [109]. Moreover, the PEGylated peptide is surrounded by a large water cloud owing to the capability of every ethylene oxide unit to bind about three water molecules [110–111]. This leads to an improved solubility and to a remarkable shielding of the peptide against proteases and antibodies [112–113] that is reflected in an improved stability and biodistribution as well as reduced immunogenicity [108]. In 1977, two key publications were reported on the PEGylation of bovine serum albumin [114] as well as the enzyme bovine liver catalase [115] with PEG of 1,900 Da and 5,000 Da, respectively, yielding in lower immunogenicity [114] and enhanced circulating lives [115]. Since then, the chemistry and application of this innovative drug delivery system has been developed crucially.

Currently, there are many strategies to incorporate PEG into peptides. They can be attached to amino groups by acylation or alkylation, to thiols, hydroxy or amide groups [108]. These polymers are commonly available with a methoxy group for endcapping at one end and a chemically activated functionality such as an active ester at the other one [109,113]. Furthermore, there is the possibility to purchase PEG differing in terms of size and molecular weight, defined by the number of chains. A conventional strategy for peptide PEGylation is the use of the SPPS technique. Here, polypeptides with specifically protected side chains are synthesized by automated SPPS according to the strategy that is applied for the introduction of fatty acids. Following deprotection of the desired side chain, this can be selectively modified with activated (mainly *N*-hydroxysuccinimidyl (NHS) esters) PEG. Small polyethylene glycol units up to 2 kDa can be introduced on the solid support [99]. The introduction of larger PEG units has to be carried out in solution. Therefore, side-chain protecting groups during the automated SPPS have to be reconsidered. If a lysine side chain is modified, the Nvoc group will have to be used to protect the N-terminus and the lysine side chains that are important for the biological functions of the peptide. After cleavage of the peptide from the resin, only the desired amino groups are available for modification. Following successful manual coupling of the PEG unit, Nvoc groups can be cleaved by UV light [43]. Using this method, a fast and automated synthesis of the peptide backbone as well as a specific modification of a peptide is possible.

It has been shown that the amino acid position plays a pivotal role for maintaining the biological functions. The incorporation of a 40 kDa branched PEG to interferon α-2a has led to one of the first launched PEGylated drugs (Pegasys^®^ [116]), which is used as an antiviral drug for the treatment of hepatitis C (Table 2) nowadays. In contrast to the solution synthesis of Pegasys^®^ via multi-PEGylation, solid-phase techniques were applied by Lee et al. [117]. They could selectively modify recombinantly produced interferon α-2a with PEGs of different sizes. Therefore, the protein was adsorbed to a cation-exchange column that served as solid matrix and PEGylation was performed at the N-terminus with 5, 10 and 20 kDa methoxy PEG-aldehydes by reductive alkylation. With this strategy, they could circumvent unspecific multi-PEGylation by maintaining the reduced immunoreactivity, which is important for hepatitis therapy [117]. As PEG has some limitations as polydispersity [109] and a lack of biodegradability [118], alternative delivery systems such as polysialylation [119], HESylation [120] or PASylation [121] are already available. However, PEGylation is still a successful depoting strategy that showed prolonged activity of various biologically active peptides (Table 2).

#### Case study: selective robot-assisted modification of NPY-receptor ligands

The 36 amino acid peptide hormones neuropeptide Y (NPY), peptide YY and pancreatic polypeptide (PP) are endogenous ligands of the so-called NPY family. They are responsible for a variety of physiological functions such as food intake, energy homeostasis, cancer, cell proliferation, blood pressure and epilepsy [122–123]. Those effects are mediated by four distinct G protein-coupled receptors (Y_1_, Y_2_, Y_4_, Y_5_) that are expressed in central and/or peripheral tissues.

**Figure 4:**
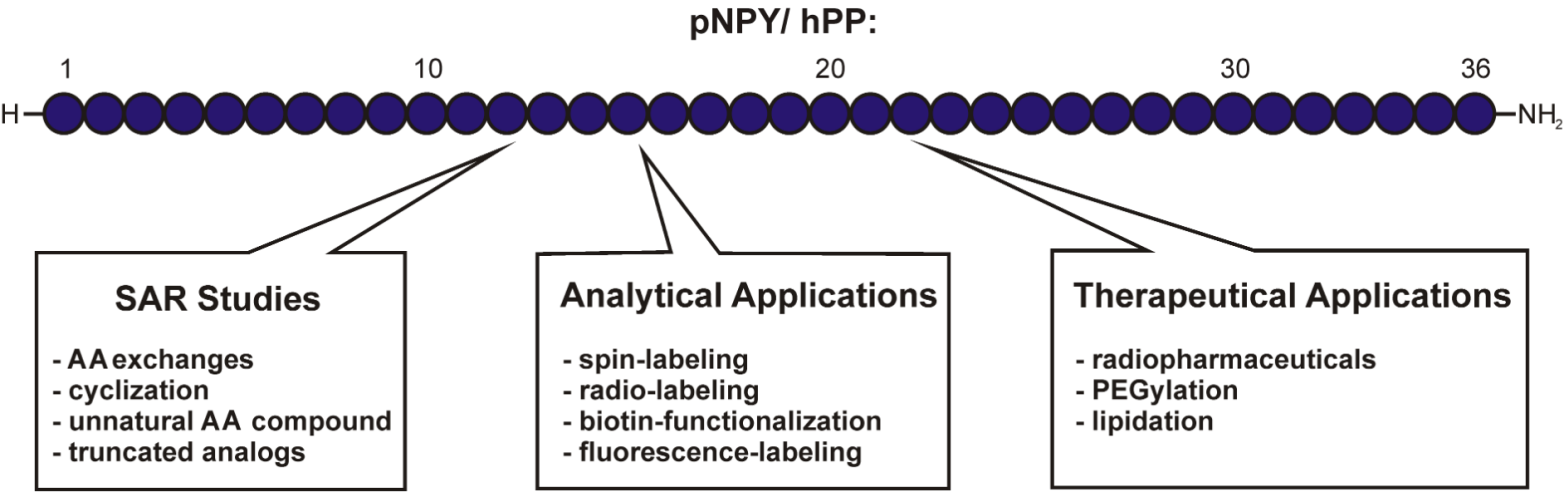
Spectrum of methods for solid phase-synthesized peptides. AA: amino acid, SAR: structure–activity relationship.

**Figure 5:**
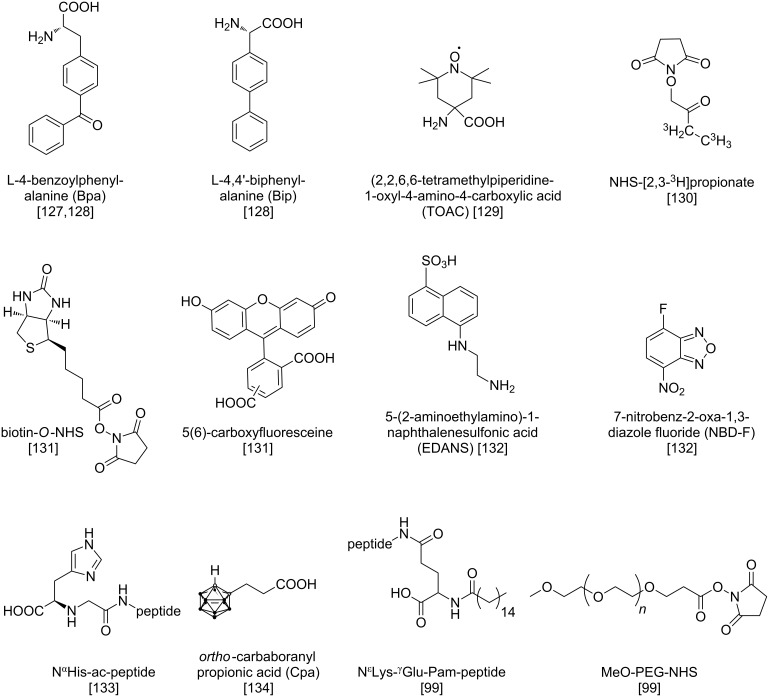
Compounds that can be introduced into pNPY (porcine neuropeptide Y) or hPP (human pancreatic polypeptide). NHS: *N*-hydroxysuccinimidyl, Pam: palmitoyl residue.

SPPS offers a great opportunity to synthetically produce these peptides in order to develop chemically engineered peptidomimetics or to uncover their distinct binding modes. Figure 4 illustrates possible strategies and Figure 5 feasible moieties for chemical modifications, which can be incorporated by semi-automated Fmoc/*t*-Bu-based SPPS. Amino acid substitutions by alanine [124] or hydrophobic/ionic monomers [125] can help to identify key binding sites of peptides and their individual receptors. In addition, it is possible to perform cyclization [126] or modification of NPY with unnatural amino acids [127]. Cabrele et al. reported on a tyrosine methyl ester linker that enabled on-resin cyclization of short segments of NPY by selective deprotection of its methyl groups after synthesis termination [126]. Besides, numerous non-canonical amino acids could be introduced by SPPS to C-terminal NPY fragments at different positions using standard coupling conditions [127]. Amino acids such as Bpa (L-4-benzoylphenylalanine) and Bip (L-4,4'-biphenylalanine) (Figure 5) were attached to the resin-bound peptides by manual coupling steps. Moreover, these first studies led to remarkably truncated NPY analogs [128]. Those structure–activity relationship (SAR) studies generated more potent and stable peptidomimetics as compared to the wild types. Most steps within the synthesis of those analogs can be performed automatically by a peptide synthesizer, providing a fast and straightforward access to the peptides.

SPPS can also be used to introduce specific tools into the peptide sequence for analytical investigations (Figure 4). These tools can be spin and radioactive labels, bioactive molecules (such as biotin) or fluorescent dyes. The paramagnetic substance TOAC (2,2,6,6-tetramethylpiperidine-1-oxyl-4-amino-4-carboxylic acid) (Figure 5) was manually coupled to NPY during SPPS allowing EPR (electron paramagnetic resonance) studies to investigate conformational changes during receptor binding [129]. Here, synthesis on solid support could be easily realized. Nevertheless, conditions for cleavage of the peptide conjugates from the resin had to be optimized owing to the sensitivity of the nitroxide group of TOAC [129]. Koglin et al. used the photo-cleavable protecting group Nvoc for selective ^3^H-labeling of full length NPY. Here, lysine residues as well as the free N-terminus of resin-bound peptide, which should not be radio-labeled, were protected with Nvoc. Following resin cleavage, radio labeling was performed with NHS-[2,3-^3^H]propionate (Figure 5) by the Bolton–Hunter reaction. Then, the Nvoc protecting groups could be removed by irradiation with UV light to obtain the fully deprotected, radio-labeled peptide [130]. Biotin and fluorophores such as CF (5(6)-carboxyfluoresceine) (Figure 5) or TAMRA (5(6)-carboxytetramethylrhodamine) can also be manually introduced to the N-terminus of NPY within the last coupling step [131]. Another work demonstrated the use of the Mtt group for selective, orthogonal side-chain protection in order to synthesize a doubly fluorescent-modified NPY analog [132]. In this study, EDANS (5-(2-aminoethylamino)-1-naphthalenesulfonic acid)-functionalized aspartic acid (Figure 5) was coupled to a N-terminal position of the peptide and after Mtt cleavage with 1% TFA, NBD (7-nitrobenz-2-oxa-1,3-diazole) was incorporated by reaction with the side chain of Dpr (α,β-diaminopropionic acid). These fluorescently labeled conjugates allowed FRET (fluorescence resonance energy transfer) measurements to investigate conformational features of the peptides [132].

With respect to the great potential of NPY ligands as pharmaceuticals for various indications such as cancer [133–134] and obesity [99], automated SPPS also offers a versatile repertoire. As an example, a method to produce radiolabeled Y_1_-receptor preferring agonists of NPY has been described, which could selectively target breast cancer cells and allowed specific tumor diagnosis. Here, the *N*^α^-histidinyl acetyl (*N*^α^His-ac) chelator (Figure 5) was attached to lysine side chains or the N-terminus of the peptide by manual coupling, whereas the chelation with ^99m^Tc occurred in solution [133]. The incorporation of carbaboranes that can be used for boron neutron capture therapy demonstrated the potential for tumor therapy as well. Here, *ortho*-carbaboranyl propionic acid (Cpa) (Figure 5) was coupled to lysine side chains on a resin-bound peptide in a manual step. An alternative Fmoc-cleavage procedure for the following coupling steps assured the stability of the piperidine-labile carbaborane moiety [134]. With respect to peptide stabilization techniques, Bellmann-Sickert et al. described a method for on-resin lipidation of hPP with palmitic acid and a strategy to synthesize PEGylated peptides [99]. For lipidation, lysine residues were protected with Dde during the automated peptide synthesis. Orthogonal deprotection of Dde enabled coupling of a glutamate spacer and the desired fatty acid (Figure 5) by amide-bond formation [99]. Synthesis of PEGylated conjugates (Figure 5) occurred on solid phase (for 2 kDa PEG) according to the same procedure. For PEGylation with a much larger group (20 kDa PEG), the N-terminus of hPP was protected with Nvoc and after cleavage from resin, selective PEGylation was performed in solution followed by specific Nvoc removal [99].

## Conclusion

The production of peptides by automated synthesis on solid support provides a great variety of benefits. Chemical reactions necessary to assemble peptides can be performed simultaneously with plenty of reaction tubes allowing parallel and multiple syntheses. Owing to the simple washing of the resin following coupling or deprotection steps, no intermediate purification is necessary. Furthermore, the possibility to use excess amounts of reagents facilitates high yields of the synthesized peptides. This fast, automated and practicable method has evolved to a major technique to produce chemically synthesized peptide therapeutics, which pushed the market steadily. Automated SPPS is of great importance, especially for structure–activity relationship studies and backbone modification of biologically active peptide hormones. Nevertheless, there are still some issues that have to be addressed. For instance, incorporation of *N*-methylated amino acids in order to improve their proteolytic stability often is difficult because of steric hindrance [135]. Moreover, the introduction of fatty acids and PEG moieties, especially large sizes, is generally performed manually [99].

Although remarkable progress has been accomplished since the invention by Merrifield more than 50 years ago [20], novel technologies in automated peptide synthesis are required. Microwave-assisted SPPS, for instance, has been shown to not only enhance reaction rates but also to enable the synthesis of difficult and rigid peptide sequences [136]. This technique is progressing but needs more improvements, especially in terms of practicability. The ongoing need for peptides as biopharmaceuticals will surely promote these developments in the future.

## References

[1] Jakubke H-D, Jeschkeit H (1982). Aminosäuren, Peptide, Proteine.

[2] Schmeing T M, Ramakrishnan V (2009). Nature.

[3] Craik D J, Fairlie D P, Liras S, Price D (2013). Chem Biol Drug Des.

[4] Bellmann-Sickert K, Beck-Sickinger A G (2010). Trends Pharmacol Sci.

[5] Vlieghe P, Lisowski V, Martinez J, Khrestchatisky M (2010). Drug Discovery Today.

[6] Bilello J A (2005). Curr Mol Med.

[7] Marx V (2005). Chem Eng News.

[8] Ahrens V M, Bellmann-Sickert K, Beck-Sickinger A G (2012). Future Med Chem.

[9] Katsila T, Siskos A P, Tamvakopoulos C (2012). Mass Spectrom Rev.

[10] Lax R (2010). The Future of Peptide Development in the Pharmaceutical Industry. PharManufacturing The International Peptide Review.

[11] (2014). U.S. Food and Drug Administration.

[12] (2014). DrugBank, Open Data Drug & Drug Target Database.

[13] McGregor D P (2008). Curr Opin Pharmacol.

[14] Frokjaer S, Otzen D E (2005). Nat Rev Drug Discovery.

[15] Fischer E (1901). Ber Dtsch Chem Ges.

[16] du Vigneaud V, Ressler C, Swan C J M, Roberts C W, Katsoyannis P G, Gordon S (1953). J Am Chem Soc.

[17] Bergmann M, Zervas L (1932). Ber Dtsch Chem Ges.

[18] Bruckdorfer T, Marder O, Albericio F (2004). Curr Pharm Biotechnol.

[19] Zompra A A, Galanis A S, Werbitzky O, Albericio F (2009). Future Med Chem.

[20] Merrifield R B (1963). J Am Chem Soc.

[21] Merrifield B (1985). Angew Chem, Int Ed Engl.

[22] Alsina J, Albericio F (2003). Biopolymers.

[23] Montalbetti C A G N, Falque V (2005). Tetrahedron.

[24] Merrifield R B (1965). Science.

[25] Fekete S, Veuthey J-L, Guillarme D (2012). J Pharm Biomed Anal.

[26] Wurtz N R, Turner J M, Baird E E, Dervan P B (2001). Org Lett.

[27] Zlatev I, Manoharan M, Vasseur J J, Morvan F (2012). UNIT 1.28 Solid-Phase Chemical Synthesis of 5′-Triphosphate DNA, RNA, and Chemically Modified Oligonucleotides. Current Protocols in Nucleic Acid Chemistry.

[28] Seeberger P H (2008). Chem Soc Rev.

[29] Green T W, Wuts P G M (1999). Protective groups in organic chemistry.

[30] Carpino L A (1957). J Am Chem Soc.

[31] Carpino L A, Han G Y (1970). J Am Chem Soc.

[32] Pennington M W, Pennington M W, Dunn B M (1995). HF Cleavage and Deprotection Procedures for Peptides Synthesized Using a Boc/Bzl Strategy. Peptide Synthesis Protocols.

[33] Meienhofer J, Waki M, Heimer E P, Lambros T J, Makofske R C, Chang C-D (1979). Int J Pept Protein Res.

[34] Anderson G W, Callahan F M (1960). J Am Chem Soc.

[35] Chang C-D, Waki M, Ahmad M, Meienhofer J, Lundell E O, Haug J D (1980). Int J Pept Protein Res.

[36] Barany G, Merrifield R B (1977). J Am Chem Soc.

[37] Atherton E, Fox H, Harkiss D, Logan C J, Sheppard R C, Williams B J (1978). J Chem Soc, Chem Commun.

[38] Beyermann M, Bienert M (1992). Tetrahedron Lett.

[39] Hsieh K-H, Demaine M M, Gurusidaiah S (1996). Int J Pept Protein Res.

[40] Bycroft B W, Chan W C, Chhabra S R, Hone N D (1993). J Chem Soc, Chem Commun.

[41] Matysiak S, Böldicke T, Tegge W, Frank R (1998). Tetrahedron Lett.

[42] Loffet A, Zhang H X (1993). Int J Pept Protein Res.

[43] Patchornik A, Amit B, Woodward R B (1970). J Am Chem Soc.

[44] Isidro-Llobet A, Álvarez M, Albericio F (2009). Chem Rev.

[45] Mergler M, Durieux J P (2005). The Bachem practice of SPPS: Tips and tricks from the experts at Bachem.

[46] Novabiochem (2012). Novabiochem: Peptide Synthesis.

[47] Bayer E (1991). Angew Chem, Int Ed.

[48] Meldal M (1992). Tetrahedron Lett.

[49] García-Martín F, Quintanar-Audelo M, Garcia-Ramos Y, Cruz L J, Gravel C, Furic R, Côté S, Tulla-Puche J, Albericio F (2006). J Comb Chem.

[50] Shelton P T, Jensen K J, Jensen K J, Shelton P T, Pedersen S L (2013). Linkers, Resins, and General Procedures for Solid-Phase Peptide Synthesis. Peptide Synthesis and Applications.

[51] Barlos K, Gatos D, Kallitsis J, Papaphotiu G, Sotiriu P, Wenqing Y, Schäfer W (1989). Tetrahedron Lett.

[52] Sieber P (1987). Tetrahedron Lett.

[53] Izdebski J, Orlowska A, Anulewicz R, Witkowska E, Fiertek D (1994). Int J Pept Protein Res.

[54] Els S, Beck-Sickinger A G, Chollet C (2010). Methods Enzymol.

[55] König W, Geiger R (1970). Chem Ber.

[56] Carpino L A (1993). J Am Chem Soc.

[57] Knorr R, Trzeciak A, Bannwarth W, Gillessen D (1989). Tetrahedron Lett.

[58] Coste J, Le-Nguyen D, Castro B (1990). Tetrahedron Lett.

[59] Itoh M (1973). Bull Chem Soc Jpn.

[60] Subirós-Funosas R, Prohens R, Barbas R, El-Faham A, Albericio F (2009). Chem–Eur J.

[61] El-Faham A, Subirós Funosas R, Prohens R, Albericio F (2009). Chem–Eur J.

[62] Houghten R A (1985). Proc Natl Acad Sci U S A.

[63] Jung G, Beck-Sickinger A G (1992). Angew Chem, Int Ed Engl.

[64] Geysen H M, Meloen R H, Barteling S J (1984). Proc Natl Acad Sci U S A.

[65] Frank R (2002). J Immunol Methods.

[66] Pedersen S L, Jensen K J, Jensen K J, Shelton P T, Pedersen S L (2013). Instruments for Automated Peptide Synthesis. Peptide Synthesis and Applications.

[67] Salisbury S A, Tremeer E J, Davies J W, Owen D E I A (1990). J Chem Soc, Chem Commun.

[68] Fox J E, Newton R, Stroud C H (1991). Int J Pept Protein Res.

[69] Lebl M, Hachmann J, Howl J (2005). High-Throughput Peptide Synthesis. Peptide Synthesis and Applications.

[70] Lebl M (2003). J Lab Autom.

[71] Collins J M, Collins M J, Steorts R C (2003).

[72] Yu H M, Chen S T, Wang K T (1992). J Org Chem.

[73] Vanier G S, Hensen K J, Shelton P T, Pedersen S L (2013). Microwave-Assisted Solid-Phase Peptide Synthesis Based on the Fmoc Protecting Group Strategy (CEM). Peptide Synthesis and Applications.

[74] Palasek S A, Cox Z J, Collins J M (2007). J Pept Sci.

[75] Lam K S, Salmon S E, Hersh E M, Hruby V J, Kazmierski W M, Knapp R J (1991). Nature.

[76] Lam K S, Lebl M, Krchňák V (1997). Chem Rev.

[77] Barlos K, Gatos D, Schäfer W (1991). Angew Chem, Int Ed Engl.

[78] Riniker B, Flörsheimer A, Fretz H, Sieber P, Kamber B (1993). Tetrahedron.

[79] Chandrudu S, Simerska P, Toth I (2013). Molecules.

[80] Greenberg M L, Cammack N (2004). J Antimicrob Chemother.

[81] Bray B L (2003). Nat Rev Drug Discovery.

[82] Shen W-C (2003). Drug Discovery Today.

[83] Veronese F M, Mero A (2008). BioDrugs.

[84] Els S, Schild E, Petersen P S, Kilian T-M, Mokrosinski J, Frimurer T M, Chollet C, Schwartz T W, Holst B, Beck-Sickinger A G (2012). J Med Chem.

[85] Linde Y, Ovadia O, Safrai E, Xiang Z, Portillo F P, Shalev D E, Haskell-Luevano C, Hoffman A, Gilon C (2008). Biopolymers.

[86] Chatterjee J, Rechenmacher F, Kessler H (2013). Angew Chem, Int Ed.

[87] Miranda L P, Holder J R, Shi L, Bennett B, Aral J, Gegg C V, Wright M, Walker K, Doellgast G, Rogers R (2008). J Med Chem.

[88] Green B R, Klein B D, Lee H-K, Smith M D, White H S, Bulaj G (2013). Bioorg Med Chem.

[89] Gentilucci L, De Marco R, Cerisoli L (2010). Curr Pharm Des.

[90] Peters T (1985). Adv Protein Chem.

[91] Kurtzhals P, Havelund S, Jonassen I, Kiehr B, Larsen U D, Ribel U, Markussen J (1995). Biochem J.

[92] Bloomfield V (1966). Biochemistry.

[93] Spector A A (1975). J Lipid Res.

[94] Ashbrook J D, Spector A A, Santos E C, Fletcher J E (1975). J Biol Chem.

[95] Curry S, Mandelkow H, Brick P, Franks N (1998). Nat Struct Biol.

[96] Simard J R, Zunszain P A, Hamilton J A, Curry S (2006). J Mol Biol.

[97] Zhang L, Bulaj G (2012). Curr Med Chem.

[98] Dasgupta P, Singh A T, Mukherjee R (1999). Pharm Res.

[99] Bellmann-Sickert K, Elling C E, Madsen A N, Little P B, Lundgren K, Gerlach L-O, Bergmann R, Holst B, Schwartz T W, Beck-Sickinger A G (2011). J Med Chem.

[100] Cheng W, Satyanarayanajois S, Lim L-Y (2007). Pharm Res.

[101] Bednarek M A, Feighner S D, Pong S-S, McKee K K, Hreniuk D L, Silva M V, Warren V A, Howard A D, Van der Ploeg L H Y, Heck J V (2000). J Med Chem.

[102] Chapman T M, Perry C M (2004). Drugs.

[103] Gallwitz B U (2007). Endocrinology.

[104] Aletras A, Barlos K, Gatos D, Koutsogianni S, Mamos P (1995). Int J Pept Protein Res.

[105] Chhabra S R, Hothi B, Evans D J, White P D, Bycroft B W, Chan W C (1998). Tetrahedron Lett.

[106] Vilsboll T (2009). Drugs Today.

[107] Léger R, Thibaudeau K, Robitaille M, Quraishi O, van Wyk P, Bousquet-Gagnon N, Carette J, Castaigne J-P, Bridon D P (2004). Bioorg Med Chem Lett.

[108] Veronese F M, Pasut G (2005). Drug Discovery Today.

[109] Veronese F M (2001). Biomaterials.

[110] Liu K-J, Parsons J L (1969). Macromolecules.

[111] Maxfield J, Shepherd I W (1975). Polymer.

[112] Israelachvili J (1997). Proc Natl Acad Sci U S A.

[113] Harris J M, Chess R B (2003). Nat Rev Drug Discovery.

[114] Abuchowski A, van Es T, Palczuk N C, Davis F F (1977). J Biol Chem.

[115] Abuchowski A, McCoy J R, Palczuk N C, van Es T, Davis F F (1977). J Biol Chem.

[116] Bailon P, Palleroni A, Schaffer C A, Spence C L, Fung W-J, Porter J E, Ehrlich G K, Pan W, Xu Z-X, Modi M W (2001). Bioconjugate Chem.

[117] Lee B K, Kwon J S, Kim H J, Yamamoto S, Lee E K (2007). Bioconjugate Chem.

[118] Knop K, Hoogenboom R, Fischer D, Schubert U S (2010). Angew Chem, Int Ed.

[119] Gregoriadis G, McCormack B, Wang Z, Lifely R (1993). FEBS Lett.

[120] Westphal M, James M F M, Kozek-Langenecker S, Stocker R, Guidet B, Van Aken H (2009). Anesthesiology.

[121] Schlapschy M, Binder U, Börger C, Theobald I, Wachinger K, Kisling S, Haller D, Skerra A (2013). Protein Eng, Des Sel.

[122] Babilon S, Mörl K, Beck-Sickinger A G (2013). Biol Chem.

[123] Pedragosa-Badia X, Stichel J, Beck-Sickinger A G (2013). Front Endocrinol.

[124] Beck-Sickinger A G, Wieland H A, Wittneben H, Willim K-D, Rudolf K, Jung G (1994). Eur J Biochem.

[125] Pedragosa-Badia X, Sliwoski G R, Dong Nguyen E, Lindner D, Stichel J, Kaufmann K W, Meiler J, Beck-Sickinger A G (2014). J Biol Chem.

[126] Cabrele C, Langer M, Beck-Sickinger A G (1999). J Org Chem.

[127] Zwanziger D, Böhme I, Lindner D, Beck-Sickinger A G (2009). J Pept Sci.

[128] Hofmann S, Frank R, Hey-Hawkins E, Beck-Sickinger A G, Schmidt P (2013). Neuropeptides.

[129] Bettio A, Gutewort V, Pöppl A, Dinger M C, Zschörnig O, Arnold K, Toniolo C, Beck-Sickinger A G (2002). J Pept Sci.

[130] Koglin N, Lang M, Rennert R, Beck-Sickinger A G (2003). J Med Chem.

[131] Fabry M, Cabrele C, Höcker H, Beck-Sickinger A G (2000). Peptides.

[132] Haack M, Beck-Sickinger A G (2009). Chem Biol Drug Des.

[133] Khan I U, Zwanziger D, Böhme I, Javed M, Naseer H, Hyder S W, Beck-Sickinger A G (2010). Angew Chem, Int Ed.

[134] Ahrens V M, Frank R, Stadlbauer S, Beck-Sickinger A G, Hey-Hawkins E (2011). J Med Chem.

[135] Teixidó M, Albericio F, Giralt E (2005). J Pept Res.

[136] Pedersen S L, Tofteng A P, Malik L, Jensen K J (2012). Chem Soc Rev.

